# The long-lasting benefits of pre-kindergarten education on autistic children’s working memory development

**DOI:** 10.1177/13623613241265996

**Published:** 2024-07-26

**Authors:** Sohyun An Kim

**Affiliations:** University of California, Los Angeles, CA, USA

**Keywords:** autism, early childhood education, executive function, pre-kindergarten, working memory

## Abstract

Working memory functions as an underlying force for school readiness, yet many autistic children have difficulties with it. Similarly, autistic children tend to start kindergarten with less school readiness compared with their peers. In addition, children from lower socioeconomic status (SES) backgrounds face additional barriers in working memory and school readiness. Preschool-age children with autism in the United States are entitled to pre-kindergarten (pre-K) education, yet it is unknown whether pre-K education produces long-lasting benefits in working memory. This study used a nationally representative data set to examine whether pre-K education has immediate and long-term benefits on the working memory development of children in the general sample, and whether it is particularly beneficial for autistic children’s working memory development, when controlling for SES. A series of multiple regression and interaction analyses indicated that, for the general sample, having attended pre-K predicted advanced working memory during the first 2 years of elementary school (K to first grade). Particularly for autistic children, the onset of such benefits started later, in Spring of first grade, but lasted longer, until Spring of third grade (3 years). Practical implications and future directions pertaining to capitalizing on autistic children’s cognitive potential and this protracted window of growth are discussed.

**Lay abstract:**

Working memory is an important skill for school success, and it involves holding information in our memory while using it to solve complex problems. However, autistic children often have difficulties with working memory tasks. Also, kindergarteners on the autism spectrum tend to be less school-ready compared with their peers. In addition, children from disadvantaged backgrounds tend to struggle more with working memory and school readiness skills. All preschool-age children on the autism spectrum in the United States are entitled to pre-kindergarten (pre-K) education. However, it is unclear whether attending pre-K helps with children’s working memory development in the long run. This study tested whether attending pre-K benefits children’s working memory development in the long run. It also tested whether pre-K is especially helpful for autistic children’s working memory development. It was found that children who attended pre-K outperformed their peers who did not attend pre-K during the first 2 years of elementary school. However, after first grade, such benefits diminished. Importantly, autistic children who attended pre-K did not demonstrate advanced working memory immediately in kindergarten, but they started to outperform their autistic peers who did not attend pre-K during first grade to third grade. This finding highlights the importance of pre-K education for autistic children in particular. It is also important for educators and parents to understand autistic children’s unique learning paths that may be different from non-autistic children. This article discusses specific ways for educators to take full advantage of the long-lasting benefits of pre-K education in autistic children’s working memory development.

## School readiness for children on the autism spectrum

Autism Spectrum Disorder (hereafter autism) is a lifelong developmental condition characterized by difficulties in social communication, presence of repetitive or restrictive behaviors, and having limited interest or play repertoire (American Psychiatric Association, 2013). While such characteristics can lead to autism-specific strengths (Bal et al., 2022; Clark & Adams, 2020; Meilleur et al., 2015), they may also impede autistic children’s school readiness and overall school adjustment in various ways. The Office of Head Start (OHS) defines school readiness as possessing the skills, knowledge, and attitude for school success, which include physical, social, and cognitive development (Office of Head Start, 2018). Research base indicates that, while school readiness is an important predictor for long-term school success (Duncan et al., 2007; Pace et al., 2019), autistic children tend to be less school-ready overall when compared with their peers (Forest et al., 2004; Marsh et al., 2017; Pellicano et al., 2017). Particularly, a recent systematic review on autistic children’s school readiness revealed that a more pronounced area of challenge for autistic children was social-emotional readiness such as self-regulation, which can affect their overall school adjustment (Marsh et al., 2017).

As defined by the OHS, school readiness encompasses a broad range of skills (Head Start, 2018), and these skills are intricately interrelated and dependent on each other (Bierman et al., 2009; Zhang & Peng, 2023). Therefore, in order to foster well-rounded school readiness for children on the autism spectrum, it is necessary to identify the skills that serve as the underlying force for multiple yet related school outcomes.

## Roles of working memory in children’s school readiness

Executive functioning (EF) is generally understood as the purposeful mechanisms that govern the operation of various cognitive processes (Miyake et al., 2000). It involves sustaining attention, resisting impulsive behaviors, mentally manipulating information, and changing course of action as needed (Diamond, 2016). Executive functioning can be broadly classified as having three subcomponents: working memory, cognitive flexibility, and inhibition (Miyake et al., 2000), although classifications may vary.

Working memory is an important component of EF, and it involves holding information mentally while adding newly incoming information, and replacing old information with updated information for relevance to the current task (Garon et al., 2008; Miyake et al., 2000; Morris & Jones, 1990). While research repeatedly finds that all components of EF are critical for well-rounded school success for all children (Aydoner & Bumin, 2023; Diamond, 2016), working memory in particular appears to have long-lasting effects on school outcomes. For instance, Ahmed and colleagues (2019) found that, out of all the EF subcomponents measured at preschool age, working memory was the only EF component that predicted working memory and academic achievements at age 15, when controlling for sociodemographic factors and other early academic readiness. More specifically, most of the early EF measures predicted academic outcomes at age 15 when other important factors such as early academic readiness and sociodemographic factors were *not* accounted for. However, when these extraneous factors were controlled for, only working memory remained as a significant predictor of later academic outcomes.

In addition to such robust longitudinal relationship, extensive research base has shown that working memory sets the stage for school readiness as evidenced by reading skills (Peng et al., 2018; Preßler et al., 2013; Rojas-Barahona et al., 2015), language acquisition (Acha et al., 2021; Roman et al., 2014), reasoning (Simms et al., 2018), mathematical skills (Bull et al., 2008; Harvey & Miller, 2017; Preßler et al., 2013), classroom engagement (Fitzpatrick & Pagani, 2012), and overall academic readiness (e.g. knowing colors, shapes, sizes, letters, and numbers) (Mann et al., 2017; Swayze & Dexter, 2018).

However, children on the autism spectrum may manifest different patterns and heightened challenges with working memory development when compared with their non-autistic peers. Specifically, while evidence indicates that autistic children tend to display greater challenges with visuospatial working memory tasks (i.e. retaining visual or spatial features) than auditory tasks (i.e. retaining speech-based information) (Kenworthy et al., 2008; Wang et al., 2017), Macizo and colleagues (2016) found that autistic students and their typically-developing peers did not differ in their visuospatial working memory capacity. In spite of such variability, findings from the current research base largely converge toward the conclusion that autistic children generally experience difficulties with both types of working memory tasks in their daily lives (Andersen et al., 2015; Chen et al., 2016; Habib et al., 2019; Happé et al., 2006; Kercood et al., 2014; Schuh & Eigsti, 2012).

Such challenges can serve as a barrier for autistic children as they transition to formal schooling, considering the impact working memory has on multiple yet closely related school readiness skills. In fact, particularly for autistic children, working memory plays a pivotal role in the development of important school-readiness skills such as social communication and behavioral readiness (Howard et al., 2023; Pellicano et al., 2017), the two core deficit areas of autism spectrum disorder as defined by the diagnostic criteria (American Psychiatric Association, 2013). In addition, working memory is found to be one of the key ingredients for social/emotional readiness such as self-regulation and self-monitored behaviors in the classroom (Savina, 2021), a common area of challenge for autistic children (Marsh et al., 2017).

The gap in working memory that exists between autistic children and their peers from their preschool years (Gong et al., 2023) implies that these children start formal schooling on an uneven playing field. Such a disparity at the onset of their schooling will likely contribute to a gap in long-term school outcomes. Promisingly, recent research studies from the past couple of decades indicate that working memory development is highly sensitive to environmental factors (Hackman et al., 2015; Hughes, 2011) and targeted interventions (Cavalli et al., 2022; Klingberg, 2010). Moreover, it is widely understood that working memory development in children generally undergoes a “sensitive period” with high malleability and susceptibility to external factors during the first five years (Garon et al., 2008). After the “sensitive period,” working memory continues to develop through childhood and early adolescence (Conklin et al., 2007; Gathercole et al., 2004; Luciana et al., 2005).

In spite of this, little is known about early contributing factors that facilitate longitudinal working memory development in autistic children. In other words, while there is a robust research base identifying working memory as an early predictor for children’s long-term school outcomes as noted earlier, far less is known about specific ways to reinforce working memory development from early ages, particularly for autistic children.

Although preliminary, there is emerging evidence that suggests possible ways to contribute to working memory development in the context of the educational environment. Approaches to learning (ATL), a set of positive learning-related behaviors (Tourangeau et al., 2019), predicted greater growth in working memory in young autistic children (S. A. Kim & Kasari, 2023b). In addition, the student–teacher relationship has been linked to working memory development for children with or without autism (S. A. Kim & Kasari, 2023b; Vandenbroucke et al., 2018). Moreover, dance instruction in a school setting as a part of the physical education (PE) curriculum (Oppici et al., 2020) and music lessons (Frischen et al., 2021) showed promising results in improving working memory performance in young children.

Considering the role of working memory in the development of various school readiness skills that are important for autistic children, exploring further ways to strengthen working memory through educational opportunities from preschool age can be a critical step in preparing autistic children for a strong start to their formal schooling. More specifically, in the United States, preschool-age children with disabilities including autism are entitled to various special education services in inclusive early childhood education (ECE) settings through Part B of the Individuals with Disabilities Education Act (IDEA, 2020). Therefore, all preschool-age (i.e. 3–5 years) autistic children in the United States are eligible for Free and Appropriate Public Education (FAPE) with an Individualized Education Program (IEP) through various types of pre-kindergarten (pre-K) programs. However, it is unclear to date if pre-K education influences long-term working memory development of autistic children in particular. Considering the importance of the early development of working memory for overall school readiness for all children, and the challenges that autistic children tend to have with their working memory, investigations of longitudinal effects of pre-K education on working memory for all children as well as autistic children specifically are highly warranted. This way, we gain a more comprehensive understanding of pre-K education’s impacts on working memory, particularly for children on the autism spectrum. Such investigation will provide clearer guidance as to how to maximize autistic children’s learning potential. In addition, research repeatedly shows that socioeconomic status (SES)-based disparity in working memory is significant from a very young age, and such gaps do not close on their own (Hackman et al., 2015; John et al., 2019; Last et al., 2018). Hence, it is crucial to control for the SES when examining children’s working memory development.

Therefore, the following research questions are proposed:

*Research Question 1 (RQ1).* Controlling for family’s SES, does pre-K education have an immediate and long-term benefit on the working memory development of school-aged children in general (i.e. full sample of children with and without disabilities)?
*Research Question 2 (RQ2).* Controlling for family’s SES, is pre-K education particularly beneficial for the working memory development of autistic children?

## Method

### Data Set

This study used the restricted version of the Early Childhood Longitudinal Study, Kindergarten Class of 2010-2011 (ECLS-K:2011) data set, sponsored by the National Center for Education Statistics (NCES) within the Institute of Education Sciences (IES) of the U.S. Department of Education. The ECLS-K:2011 data set is a nationally representative data set, which follows the same cohort of children from kindergarten through fifth grade in the United States (Tourangeau et al., 2019). Data were collected from Fall of kindergarten in the year 2010 (T1) through Spring of fifth grade in the year 2016 (T9), across nine time points (Table 1). The public-use data files can be accessed through https://nces.ed.gov/ecls/dataproducts.asp. This study is approved for a Certified Exempt status by the Institutional Review Board (IRB#23-001153) at the author’s affiliated institution.

**Table 1.** Data collection schedule from T1 to T9.

	Semester and Grade	School year
T1	Fall of kindergarten	2010–2011
T2	Spring of kindergarten	
T3	Fall of first grade	2011–2012
T4	Spring of first grade	
T5	Fall of second grade	2012–2013
T6	Spring of second grade	
T7	Spring of third grade	2014
T8	Spring of fourth grade	2015
T9	Spring of fifth grade	2016

Source: U.S. Department of Education, National Center for Education Statistics (NCES), The Early Childhood Longitudinal Study, Kindergarten Class of 2010-2011 (ECLS-K:2011). Restricted-use data files.

### Participants

In total, approximately 18,170 children participated in the data collection, and the entire sample was included in the current analysis. That is, all children who participated in the data collection regardless of their disability status (i.e. full sample) were included in the analytic sample.

Of those, approximately 310 students were identified as having autism. In T2, T4, T6, T7, T8, and T9, parents were asked during the parent interview, “Did you obtain a diagnosis of a problem from a professional?” If the response was *yes*, they were asked a follow-up question to specify what the diagnosis was. One of the options was *autism spectrum disorder (ASD).* In addition, special education teachers were asked in T2, T4, T6, T7, T8, and T9 if the child was receiving special education or related services for a diagnosis of autism. The autism variable was assigned a value of 1 if (1) parents responded at least once during the six rounds of interviews that the child had a diagnosis of ASD or (2) the special education teacher responded at least once that the child was receiving special education services for a diagnosis of autism. If not, 0 was assigned. The autism variable was used to create an interaction term to determine if pre-K education impacted autistic children’s working memory differently than it did for the general sample (i.e. full sample).

The sample size is rounded to the nearest 10 as per the confidentiality agreement.

### Measures

#### Demographic characteristics

Demographic characteristics of the sample included (1) race/ethnicity (White, Black, Hispanic, Asian-American/Pacific Islanders/Native Americans (AAPINA), other), (2) sex assigned at birth (male, female), (3) income range, and (4) parents’ educational level.

#### Variables

##### Working memory

In the ECLS-K:2011 data set, participating children’s auditory working memory was measured. Auditory working memory capacity is commonly measured with memory span tasks. Memory span tasks typically require ordered serial recall of a sequence, and the length of the sequence correctly recalled is used as a measure for working memory capacity. Examples of memory span tasks include forward and backward digit span such as Numbers Reversed subset from the Woodcock-Johnson III (WJ III: Woodcock et al., 2001) or N-Back tasks (Owen et al., 2005). For the purpose of the ECLS-K:2011 data collection, Numbers Reversed subset from the Woodcock-Johnson III (WJ III: Woodcock et al., 2001) was used to measure working memory. Data were collected across nine time points from kindergarten to fifth grade. Children were asked to repeat auditorily presented numbers in reverse order, starting with two-number sequences. Five two-number sequences were presented before progressing to three-number sequences. The length of sequence increased after five trials, up to a maximum of eight numbers. If the child responded incorrectly for three consecutive trials, the task ended instead of progressing to a longer number sequence. Each item was scored as “correct,” “incorrect,” or “not administered” (Tourangeau et al., 2019).

For the current study, the *W* score was used. The *W* score is a standardized equal-interval score that represents both a child’s ability and the item’s difficulty. It is particularly suited for longitudinal analyses, regression, and correlation (Tourangeau et al., 2019).

In order to examine the immediate effect of pre-K education on working memory, data from T1 (Fall of kindergarten) was used as dependent variables. In addition, to investigate the longitudinal effect of pre-K education, data from T4 (Spring of first grade), T6 (Spring of second grade), T7 (Spring of third grade), T8 (Spring of fourth grade), and T9 (Spring of fifth grade) were used as dependent variables.

Missingness in the autism sample ranged from 21% to 38% while missingness in the entire sample was approximately 10%. The higher rate of missingness in the autism sample was hypothesized to be due to some of these children not being able to participate in the assessment (e.g. their Individualized Education Plan (IEP) precluding them from taking the assessment). Missing data were handled by listwise deletion.

##### Pre-kindergarten education

In T1, parents were asked during the parent interview, “Did (child) attend a daycare center, nursery school, preschool, or prekindergarten program *on a regular basis the year before* (he or she) started kindergarten?” If the parents responded *yes*, a value of 1 was assigned. If not, 0 was assigned.

##### Sex assigned at birth (hereinafter sex)

A composite variable for students’ sex was drawn from parent-reported information about the child’s sex. When the parent data were not available, the school’s administrative record was used.

##### Race

A composite variable for students’ race was drawn from either the parent-reported information or the school’s administrative records. The administrative records were used only if parent responses about the child’s race were missing.

##### Socioeconomic Status (SES)

Student’s SES was computed three times during the data collection period, at T1, T4, and T9 by the NCES, the sponsor of the ECLS-K data set. It was computed using responses from the parent interview. The five components used to create the SES variable were (1) parent 1’s education, (2) parent 2’s education, (3) parent 1’s occupation, (4) parent 2’s occupation, and (5) household income. Not all parents responded to every question, and missing data were imputed using longitudinal imputation and hot deck imputation. After the imputation, a composite value was computed for each case, and the *z*-scores of these values were used (Tourangeau et al., 2019). For more details on how this variable was computed, readers should refer to the User’s Manual (Tourangeau et al., 2019). Students’ SES data from T1 were used for the current analysis.

### Analyses

To test whether pre-K education influences children’s working memory performance immediately upon entering kindergarten as well as throughout their elementary school years, six separate multiple regression and interaction analyses were conducted with the following variables as the dependent variables: working memory at T1 (Fall of kindergarten), T4 (Spring of first grade), T6 (Spring of second grade), T7 (Spring of third grade), T8 (Spring of fourth grade), and T9 (Spring of fifth grade). Due to a multicollinearity issue among the dependent variables, it was decided that MANOVA or MANCOVA was not suitable for the current analysis. Instead, separate multiple regressions were conducted to determine statistical significance. Bonferroni correction was applied in order to reduce the possibility of Type 1 errors due to multiple comparisons. With these six comparisons, *p*-values less than 0.00833 were considered statistically significant throughout this study.

The following variables entered each model in the following order hierarchically in two blocks: (1) *sex, race*, and *SES*, and (2) *autism, pre-k education*, and autism*pre-k education interaction term. Children’s *sex, race*, and *SES* were included in the model for a controlling purpose, and *autism* and *pre-k education* entered the model to test their predictive powers on children’s working memory performance. The interaction term entered the model to test whether having autism moderates the relationship between pre-K education and working memory development.

Assumptions for multiple regression were tested with the predictor variables that entered each of the six models. To test the assumption of the linear relationship between the IVs and the DV, a scatter plot was generated. Upon visual analysis, no violation of assumption was detected. In order to test the assumption that there are no multicollinearities in the data, an analysis of collinearity statistics was conducted for each model. VIF scores were well below 10, ranging between 1.000 and 3.072. Furthermore, the tolerance scores were all above 0.2, ranging from 0.325 to 1.000.

To test the assumption of independent residuals, the Durbin-Watson statistic was conducted. The Durbin-Watson statistics ranged from 1.968 to 2.005. To test the assumption that the residuals are normally distributed, a P-P plot for each of the six models was generated. The P-P plots suggested that the assumption of normality had been met. Finally, to test the assumption that there are no influential cases biasing the models, Cook’s distance was calculated. Cook’s Distance values were all under 1 for all models (maximum = 0.03657), suggesting individual cases were not unduly influencing the models. In summary, no violations of these assumptions were found in any of the models. The above analyses were conducted using SPSS version 27.

### Community involvement

A parent of an autistic child developed the outline of the introduction section together with the author. This community member reviewed the research questions and results of this study, and acknowledged the study’s significance to the autistic community.

## Results

### Demographic characteristics

**Figure 1.**
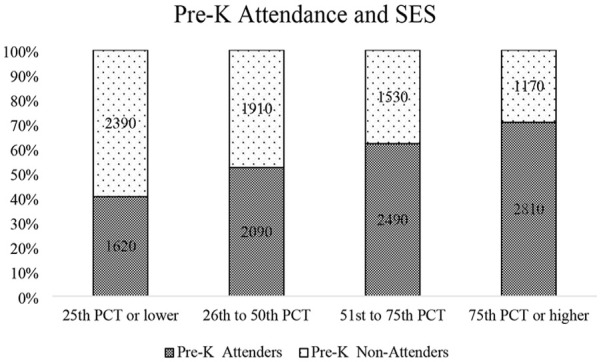
Relationship between socioeconomic status and pre-K attendance. *Source.* U.S. Department of Education, National Center for Education Statistics (NCES), The Early Childhood Longitudinal Study, Kindergarten Class of 2010-2011 (ECLS-K:2011). Restricted-use data files. *N* rounded to the nearest 10 per confidentiality agreement; percentages are rounded to the nearest whole number. *Higher percentile means higher SES score.

The demographic characteristics of the sample are illustrated in Table 2. The majority of the children in the sample were White (47%). Twenty-nine percent of the parents attended 2- to 4-year colleges, and 51% of the children were male. In addition, children from higher SES backgrounds were more likely to have received pre-K education than those from lower SES backgrounds. Table 3 and Figure 1 illustrate the relationship between SES and pre-K attendance.

**Table 2.** Demographic characteristics of the sample.

Demographic Characteristics	(*N* = 18,170)
Race	
White	8490 (47%)
Black/African-American	2400 (13%)
Hispanic	4590 (25%)
Asian-American/Pacific Islanders/Native American	1830 (10%)
Other	830 (5%)
Sex Assigned at Birth	
Female	8890 (49%)
Male	9290 (51%)
Income	
US$20,000 or less	2730 (15%)
US$20,000 to US$30,000	1750 (10%)
US$30,000 to US$50,000	2250 (12%)
US$50,000 to US$75,000	2190 (12%)
US$75,000 to US$100,000	1760 (10%)
US$100,000 to US$200,000	2260 (12%)
US$200,000 or more	590 (3%)
Parents’ educational level	
High school	3360 (19%)
2- to 4-year college	5260 (29%)
Postgraduate degree	1600 (9%)

Source: U.S. Department of Education, National Center for Education Statistics (NCES), The Early Childhood Longitudinal Study, Kindergarten Class of 2010-2011 (ECLS-K:2011). Restricted-use data files.

*N* rounded to the nearest 10 per confidentiality agreement.

**Table 3.** Relationship between socioeconomic status and pre-K attendance.

SES Quartiles*	≤ 25th percentile	26th to 50th percentile	51st to 75th percentile	≥ 75th percentile	Total
Non pre-K attenders	2390 (60%)	1910 (48%)	1530 (38%)	1170 (29%)	7000 (44%)
Pre-K attenders	1620 (40%)	2090 (52%)	2490 (62%)	2810 (71%)	9000 (56%)
Total	4010 (100%)	4000 (100%)	4020 (100%)	3980 (100%)	16,010 (100%)
Pearson χ^2^ = 825.428; *p* < 0.001					

Source: U.S. Department of Education, National Center for Education Statistics (NCES), The Early Childhood Longitudinal Study, Kindergarten Class of 2010-2011 (ECLS-K:2011). Restricted-use data files.

N rounded to the nearest 10 per confidentiality agreement; percentages are rounded to the nearest whole number.

* Higher percentile means higher SES score.

### Predictors

#### Pre-kindergarten education

**Figure 2.**
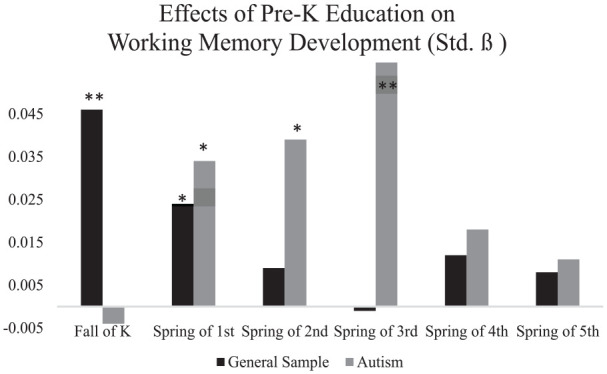
Effects of pre-K education on children’s working memory development over time Source: U.S. Department of Education, National Center for Education Statistics (NCES), The Early Childhood Longitudinal Study, Kindergarten Class of 2010-2011 (ECLS-K:2011). Restricted-use data files. *<0.00833 (significant at *p* < 0.00833 with Bonferroni correction). **< 0.001.

Table 4 and Figure 2 summarize the results of multiple regression analyses for all six time points. For all children in general, having received pre-K education the year before kindergarten predicted advanced working memory at T1 (Fall of kindergarten) (Std. ß = 0.046, *p* < 0.001) and T4 (Spring of first grade) (Std. ß = 0.024, *p* = 0.004). However, the effect of pre-K education on children’s working memory subsided after first grade (*p* > 0.00833).

**Table 4.** Multiple regression models summary for each time point.

Time Point	Variables	Beta	*t*	Sig.	Cohen’s D	ES Interpretation
T1 (Fall of K)	Constant	430.33	828.60	<0.001**		
	Race (White = 1)	6.99	14.04	<0.001**		
	Sex (Male = 1)	–2.18	–4.58	<0.001**		
	SES	10.97	34.85	<0.001**		
	Autism	–12.70	–3.63	<0.001**		
	Pre-K	2.89	5.64	<0.001**	0.095	very small
	Autism*Pre-K	–1.14	–0.27	0.789	–0.005	no effect
	*R* ^2^	0.134				
	*F*	361.73	(*p* < 0.001)			
T4 (Spring 1st Gr.)	Constant	468.56	1076.38	<0.001**		
	Race (White = 1)	4.45	9.99	<0.001**		
	Sex (Male = 1)	–1.84	–4.36	<0.001**		
	SES	7.34	26.51	<0.001**		
	Autism	–29.58	–11.63	<0.001**		
	Pre-K	1.27	2.86	0.004*	0.048	very small
	Autism*Pre-K	8.93	2.68	0.007*	0.045	very small
	*R* ^2^	0.09				
	*F*	236.36	(*p* < 0.001)			
T6 (Spring 2nd Gr.)	Constant	480.84	1161.36	<0.001**		
	Race (White = 1)	2.44	5.75	<0.001**		
	Sex (Male = 1)	–1.09	–2.73	0.006*		
	SES	5.67	21.59	<0.001**		
	Autism	–27.34	–11.56	<0.001**		
	Pre-K	0.43	1.02	0.306	0.017	no effect
	Autism*Pre-K	9.15	2.94	0.003*	0.050	very small
	*R* ^2^	0.07				
	*F*	151.05	(*p* < 0.001)			
T7 (Spring 3rd Gr.)	Constant	490.65	1186.92	<0.001**		
	Race (White = 1)	1.56	3.69	<0.001**		
	Sex (Male = 1)	–1.50	–3.78	<0.001**		
	SES	5.93	22.66	<0.001**		
	Autism	–27.87	–11.85	<0.001**		
	Pre-K	–0.05	–0.13	0.90	–0.002	no effect
	Autism*Pre-K	12.46	4.06	<0.001**	0.069	very small
	*R* ^2^	0.07				
	*F*	149.38	(*p* < 0.001)			
T8 (Spring 4th Gr.)	Constant	497.95	1189.75	<0.001**		
	Race (White = 1)	0.93	2.17	0.03		
	Sex (Male = 1)	–1.39	–3.45	<0.001**		
	SES	5.85	22.12	<0.001**		
	Autism	–19.99	–8.39	<0.001**		
	Pre-K	0.52	1.23	0.217	0.021	no effect
	Autism*Pre-K	3.76	1.22	0.224	0.021	no effect
	*R* ^2^	0.07				
	*F*	131.58	(*p* < 0.001)			
T9 (Spring 5th Gr.)	Constant	503.85	1131.27	<0.001**		
	Race (White = 1)	0.83	1.83	0.068		
	Sex (Male = 1)	–0.83	–1.95	0.052		
	SES	6.60	23.57	<0.001**		
	Autism	–21.56	–8.50	<0.001**		
	Pre-K	0.38	0.84	0.4	0.014	no effect
	Autism*Pre-K	2.44	0.74	0.461	0.012	no effect
	*R* ^2^	0.08				
	*F*	143.58	(*p* < 0.001)			

Source: U.S. Department of Education, National Center for Education Statistics (NCES), The Early Childhood Longitudinal Study, Kindergarten Class of 2010-2011 (ECLS-K:2011). Restricted-use data files.

** < 0.001.

* <0.00833 (significant at *p* < 0.00833 with Bonferroni correction).

#### Autism

Having autism predicted lower working memory at all time points, starting from Fall of kindergarten (Std. ß = −0.050, *p* < 0.001) to Spring of fifth grade (Std. ß = −0.125, *p* < 0.001).

#### Pre-kindergarten education on working memory development: autistic children

Particularly for autistic children who received pre-K education, the onset of pre-K education’s positive predictive power on working memory started later, in Spring of first grade (T4) (Std. ß = 0.034, *p* = 0.007), and such effects lasted longer, until Spring of third grade (Spring of third grade) (Std. ß = 0.057, =< 0.001).

## Discussion

This study explored the longitudinal effects of pre-K education on the working memory development of school-aged children in general, and examined whether pre-K education would be particularly beneficial for children on the autism spectrum. Generally, children who attended pre-K displayed advanced working memory (i.e. larger working memory capacity) upon school entry (i.e. Fall of kindergarten). However, such benefits faded out after 2 years of their schooling. In other words, pre-K attenders in the general sample did not perform significantly better or worse than pre-K non-attenders on their working memory tasks after Spring of first grade. Such “fade-out” phenomena have been documented with several studies that examined the longitudinal effects of preschool education for the general sample (Abenavoli, 2019; Burchinal et al., 2022; Puma et al., 2012; Yoshikawa et al., 2016). Interestingly though, particularly for autistic children, the effects of pre-K education on working memory did not emerge immediately upon school entry in Fall of kindergarten. Yet, autistic children who received pre-K education started to outperform their autistic peers who did not attend pre-K on the working memory tasks, starting from Spring of first grade, and such advantage lasted until Spring of third grade, which is 2 years after the effects of pre-K faded out for the general sample. Therefore, it appears that the benefits of pre-K education “kick in” later for autistic children, while such benefits last longer when compared with the general sample. The current finding partly aligns with the previous finding which indicated that some autistic students, particularly those who started with poor working memory upon school entry (i.e. “late-bloomers”), did not start to show rapid growth in working memory until the later years of their elementary school. However, these “late-bloomers” showed greater growth in working memory in their late elementary school years, which was when their neurotypical peers slowed down in their working memory development (S. A. Kim & Kasari, 2023a). Nevertheless, factors that may have contribute to such “late-blooming” effects had been unclear to date. Moreover, no prior studies examined the role of pre-K education on autistic children’s longitudinal working memory development over the entire elementary school period. This study contributes to the literature base by demonstrating that attending a pre-K program can be one important way to foster greater growth in working memory particularly for autistic children, which can set them up for success in their long-term school outcomes.

### Pre-K benefits on working memory development

It appears that, for the general sample, various environmental factors other than pre-K education may have contributed to their working memory development as they continued their schooling in their elementary schools. However, for autistic children, pre-K education appears to have a slower but more robust influence on their long-term working memory development.

The delayed onset of the pre-K benefits on autistic children’s working memory development may be explained by the heavy language requirement of this specific measurement tool (i.e. Numbers Reversed subset from the Woodcock-Johnson III). It is estimated that approximately 90% of autistic children experience varying levels of language delays (Nicholas et al., 2008). Thus, this instrument may not be sensitive enough to capture the pre-K benefits on working memory for autistic children with language delays in their early years. In other words, these children’s “true gains” in working memory may not have been reflected in the scores they received on this particular instrument until their language skills improved. Future studies must test if a similar phenomenon is observed when their working memory gains are measured through various instruments with less language load, or if visuospatial working memory is measured instead of auditory working memory. For example, children’s everyday working memory can be measured by the BRIEF through a parent questionnaire (Gioia et al., 2000), and visuospatial working memory can be measured by the Cambridge Automated Neuropsychological Test Battery (CANTAB) (Smith et al., 2013). If a similar phenomenon is indeed observed, it would be worthwhile to investigate the underlying mechanisms that result in such delayed onset of pre-K effects on autistic children’s working memory development.

While this specific aspect of the instrument may explain the delayed onset of the pre-K benefits displayed in the study, the long-lasting effect of pre-K education on autistic children’s working memory was encouraging yet unexpected. No prior studies were found that investigated the benefit of pre-K education on autistic children’s longitudinal working memory development in particular. However, the long-lasting effects of pre-K education on autistic children’s working memory can potentially be explained by earlier studies on young autistic children’s brain development. Research studies on early brain development indicate that intrinsic factors such as genetic information as well as external factors such as repeated experiences and interactions with the environment, together facilitate cognitive development in very young children (Johnson & Munakata, 2005; Shonkoff, 2003). Hence, children’s early and naturally occurring interactions with their social environment play a key role in their brain development in typical scenarios. However, such experience-expectant learning may not occur naturally for autistic children as these children interact with their social environment differently. Studies show that autistic children show atypical motivation for social stimuli from their first year of life (Dawson et al., 2005; Stavropoulos & Carver, 2014; Zwaigenbaum et al., 2005), which can potentially interfere with their experience-expectant learning and brain development. Thus, it can be hypothesized that early exposure to positive and structured social engagements through pre-K education may have helped autistic children to be more receptive to social experiences, which in turn would have facilitated effective learning. This can also explain the less robust effect of pre-K education on the general sample: the general sample’s brain development can be facilitated through naturally occurring experiences with or without structured social stimulation; therefore, pre-K education may have played a less salient role.

Moreover, pre-K education may have provided early exposure to a learning environment that reinforces behaviors related to advanced ATL and positive student–teacher relationship, which are linked to advanced working memory for autistic children (S. A. Kim & Kasari, 2023b; Vandenbroucke et al., 2018). Taken together, it is hypothesized that socially rich learning environments from preschool age may generate more robust benefits for autistic children whose experience-expectant learning is not as effective as the general population.

### Implications and recommendations

The current study directs us to look beyond the intrinsic autism characteristics to recognize the role of the learning environment in preschool ages that influences their working memory development. Although the current study did not parse out what type of program these children attended (e.g. inclusive, special education, state-funded, private), the dosage (e.g. full-day, half-day, and number of days per week), or what skills were targeted through these programs, it provides us with promising evidence that participating in school-based programs prior to entering kindergarten may produce long-lasting benefits in working memory development for autistic children. While all children with disabilities ages 3 to 5 in the United States should have access to high-quality inclusive early childhood education (ECE) through Part B of IDEA, only approximately 40% of preschool-aged children with developmental disabilities are receiving such services (CDC, 2022; US DOE, 2020). This may be due to delayed identification of disabilities and therefore a missing out on the window. Recent data show that autistic children’s median age at first diagnosis was 51 months (Maenner et al., 2020), and such delays were more prevalent among children from low SES backgrounds (Fountain et al., 2011; Mazurek et al., 2014; Parikh et al., 2018) and those from traditionally minoritized ethnic backgrounds (Wiggins et al., 2020). Such disparities would prevent autistic children from minoritized backgrounds from taking full advantage of the ECE programs through Part B of IDEA. Therefore, timely identification of autism through universal screening and accessing appropriate services would be an important first step in maximizing these children’s cognitive potential. Other challenges to timely access to pre-K education may include barriers in navigating the education system, or complications relating to the diagnostic decisions when the disability intersects with other minoritized statuses such as speaking languages other than English (Stahmer et al., 2019). Future studies must further explore challenges that families with autistic children face when accessing pre-K education and other related services and ways to alleviate those challenges.

In addition to increasing access to pre-K programs for autistic children, the characteristics of the pre-K programs that are associated with greater gains must also be taken into serious consideration. Pre-K programs vary in terms of type and quality, resulting in varying levels of benefit in children’s development. Such variability may lead to confusion when parents make decisions on their young autistic children’s pre-K placement. One important question parents may have is whether to choose an inclusive setting or a specialized setting exclusively for children with autism or other developmental disabilities (Byrne, 2013; Samadi & McConkey, 2018). To answer this question, Nahmias and colleagues (2014) compared the cognitive gains of preschool children on the autism spectrum based on their educational placement type. After controlling for the initial cognitive and demographic characteristics, it was concluded that inclusive preschool learning environments were superior to disability-only environments in facilitating greater cognitive outcomes in autistic children. Furthermore, inclusive programs often score higher than restrictive settings on the quality measure (Weglarz-Ward & Santos, 2018). Successful inclusion in preschool settings is linked to the following quality indicators: (1) effective integration of interventions into instructions (Weglarz-Ward et al., 2019), and (2) a school culture that reinforces close collaboration with interventionists and educators (Weglarz-Ward et al., 2020), as well as active parent participation (Chan & Ritchie, 2016). Such information could be helpful for parents when they are searching for high quality inclusive pre-K education for their autistic child.

Moreover, some parents from culturally and linguistically diverse backgrounds tend to be hesitant to expose their autistic child to mainstream environments due to the stigma around disabilities and negative comments from their own community members, typically arising from their misunderstanding of autism (e.g. family members blaming the mother for the child’s autism) (H. Kim et al., 2023). Therefore, increasing awareness on autism and inclusivity through peer support groups, community events and parent training is essential in eliminating the stigma. Consequently, such efforts will contribute to increasing their willingness to participate in inclusive pre-K education.

One important but less explored barrier to inclusive pre-K programs is the perspectives of parents of children without disabilities about participating in inclusive pre-K programs (Siller et al., 2021). Although inclusive learning environments are found to be beneficial for children without disabilities, especially in the areas of empathy and acceptance (Kart & Kart, 2021), information on the parents’ perspectives remain mixed or unclear (Hilbert, 2014; Su et al., 2020; Vlachou et al., 2016). Therefore, future studies must continue to explore the benefits of inclusive environments specifically for children without disabilities, and to examine parents’ feelings toward their children’s participation in inclusive programs. More importantly, a raised awareness of the benefits of inclusive pre-K for children without disabilities must be prioritized through dissemination of evidence.

Current findings also have implications for teachers and parents in that “cognitive endurance” must be promoted when educating children on the autism spectrum. It may be erroneously perceived that autistic children are making limited progress if such a conclusion is based on their short-term gains, which can potentially lead to learned helplessness (Seligman, 1972). However, the above findings demonstrate that autistic children may be undergoing a pattern of working memory development that is different from their non-autistic peers. In other words, autistic children may be making slower but more steady progress in working memory, and pre-K education may be producing a long-lasting and robust influence on autistic children’s working memory development through socially enriched learning environments. Learned helplessness could be alleviated by implementing behavior-analytic strategies such as providing immediate positive reinforcement, behavior-specific feedback, a personalized and supportive learning environment, and helping to set short- and long-term goals with defined rewards (Ghasemi & Karimi, 2021). That way, children on the autism spectrum can follow their own unique developmental path and maximize their potential without experiencing the detrimental effects of learned helplessness.

## Limitations and future directions

A few limitations must be noted in this study. First, the findings from the study must be interpreted with caution due to the higher rate of missingness in the autism sample’s working memory variable compared with the entire sample. In other words, since some of the missingness is unlikely to be due randomness (e.g. their IEP precluding them from taking an assessment), the findings may not be representative of the entire spectrum of autistic children. In addition, as previously mentioned, the current analysis did not account for the types of pre-K programs (e.g. Head Start, state-funded, private), the setting (e.g. inclusive, special education), teacher practices, skills taught, dosage, or other school-level factors that could have contributed to the longitudinal effect on children’s working memory development. This was in fact an intentional choice for the following reason: The ECLS-K:2011 data set used longitudinal data by following the same group of children from 2011 to 2016, from kindergarten to fifth grade, which indicates that these children attended their pre-K programs in 2010–2011. There have been many important changes in the ECE landscape in the past decade or so in terms of the enrollment (OECD, 2023; UNICEF, 2022), demographics, and curriculum and instruction (Haslip & Gullo, 2018), particularly due to the COVID-19 pandemic (Ackerman & Friedman-Krauss, 2017; Friedman-Krauss et al., 2022). Therefore, parsing out specific features that could be more or less beneficial would be less relevant or meaningful. Instead, this study broadly examined the benefits of exposing young children to educational and socially engaging environments prior to entering kindergarten. Therefore, it is unknown from the current findings which specific features of pre-K programs were predictive of advanced working memory. A new cohort of children who started kindergarten in 2023–2024 is being studied by the IES, and an updated data set (ECLS-K:2024) will be released in the future. Future studies must engage in in-depth evaluations of the individual effects of each of the aforementioned factors in pre-K education on children’s working memory development when the new data set is released. That way, the implications drawn from the study will be more relevant to the current educational landscape. Moreover, due to the nature of a secondary data analysis, the autism sample in the current study was based on parental report in the data set, and no medical diagnosis was confirmed, and the severity of the autism presentations were not reported. Furthermore, this study only explored one cognitive outcome, working memory, while no single cognitive measure can be a valid representation of the holistic benefits that pre-K education may bring forth. Therefore, other important school outcomes such as literacy skills, mathematical skills, social and emotional readiness as well as other executive functioning skills must be explored in order to gain a comprehensive understanding of the effects of pre-K education on autistic children. More importantly, future studies must explore how autistic children’s gains in working memory through pre-K education later transfer to other academic skills. Finally, although children’s SES was used as a controlling variable in the current analysis, and therefore was not interpreted substantively in this study, SES was a significant predictor for children’s working memory at all time points. Such SES-based disparities in working memory are well-documented in the existing research base and this gap does not close on its own (Hackman et al., 2015; John et al., 2019). Future studies must continue to investigate specific ways to interrupt this gap. Universal access to high-quality pre-K education for all children from low SES backgrounds with and without disabilities in inclusive settings through state-funded programs can be particularly instrumental in closing such a gap. Accessing early intervention services for young toddlers ages 0 to 3 through Part C of IDEA can also be an effective way to equip autistic children from low SES backgrounds with the necessary skills to be successful in their pre-K education and beyond.
